# Early pregnancy cardio metabolic risk factors and the prevalence of metabolic syndrome 10 years after the first pregnancy

**DOI:** 10.1371/journal.pone.0280451

**Published:** 2023-01-20

**Authors:** Prabha H. Andraweera, Michelle D. Plummer, Amy Garrett, Shalem Leemaqz, Melanie R. Wittwer, Emily Aldridge, Maleesa M. Pathirana, Gus A. Dekker, Claire T. Roberts, Margaret A. Arstall

**Affiliations:** 1 Adelaide Medical School, The University of Adelaide, Adelaide, Australia; 2 Robinson Research Institute, The University of Adelaide, Adelaide, Australia; 3 Department of Cardiology, Lyell McEwin Hospital, Elizabeth Vale, Australia; 4 Flinders Health and Medical Research Institute, Flinders University, Bedford Park, SA, Australia; 5 Division of Women’s Health, Lyell McEwin Hospital, Elizabeth Vale, Australia; University of the Witwatersrand, SOUTH AFRICA

## Abstract

**Background:**

We aimed to compare risk factors for CVD 10 years postpartum among women who had ≥ 1 compared to no cardio metabolic risk factor in early first pregnancy.

**Methods:**

Women of the SCOPE (Screening fOr Pregnancy Endpoints) study from Adelaide, South Australia were invited to participate in a cardiovascular risk assessment 10 years after the delivery of the first child. Data from 141 women who completed all the assessments are included in the analyses.

**Result:**

Compared to women who did not have any cardio metabolic risk factor at 15 ± 1 weeks’ gestation during the first pregnancy, those who had ≥ 1 risk factor were 5.5 times more likely to have metabolic syndrome 10 years postpartum (aOR = 5.5, 95% CI 1.8–17.3, p = 0.004). Women who had ≥ 1cardio metabolic risk factor during the first pregnancy were more likely to be obese (p = 0.001), have high total cholesterol levels (p <0.001) or have increased insulin resistance (p <0.001) 10 years later compared to women who had no risk factor during the first pregnancy. 63.5% of the women with no cardio metabolic risk factor compared to 39% of women who had ≥ 1 risk factor in first pregnancy, had neither a complicated first pregnancy nor was diagnosed with MetS 10 years postpartum (p = 0.023).

**Conclusion:**

Cardio metabolic risk factors at the booking visit in the first pregnancy may be useful in identifying young women at risk of future CVD.

## Introduction

The past few decades have shown marked improvements in cardiovascular disease (CVD) outcomes. However, these trends have not been consistent across all populations [1]. The incidence of acute myocardial infarction among young women has been increasing, largely due to the increased global prevalence of conventional risk factors including obesity, hypertension and type 2 diabetes mellitus [2]. Data from the United States demonstrates that the annual death rate attributed to coronary heart disease among women fell by 4.4% from 2000 to 2002, but increased by 1.5% among women aged 35–54 years [2]. In addition, a history of major pregnancy complications, including gestational hypertension, preeclampsia, gestational diabetes mellitus, stillbirth, preterm birth, placental abruption, or intrauterine growth restriction is also more likely to be reported among women who are diagnosed with coronary heart disease [3]. The consistent evidence that links these pregnancy complications with CVD has been acknowledged by many professional societies including the American Heart Association, the American College of Obstetricians and Gynecologists, and the Society of Obstetricians and Gynaecologists of Canada. A recent advisory committee from the American Heart Association and the American College of Obstetricians and Gynecologists recommended the need for targeted screening and long term follow up of these women as part of routine postpartum care [4].

Cardiovascular risk stratification based on major pregnancy complications is likely to identify many women at risk of CVD at a young age. However, the fact that pregnancy complications may not be causal of CVD but rather markers of an underlying predisposition, that itself is associated with the development of major pregnancy complications and CVD needs to be considered. In fact, both pregnancy complications and CVD share many risk factors including, genetics and modifiable environmental and lifestyle factors. Therefore, it can be proposed that some young women may actually have pre-existing cardio-metabolic risk factors and hence would be at increased risk of developing CVD irrespective of experiencing a major pregnancy complication [5].

Metabolic syndrome (MetS), a collection of metabolic and vascular risk factors is an established risk for coronary heart disease. A meta-analysis of data from 951, 083 patients demonstrated that MetS is associated with two-fold increased risk of CVD, CVD mortality, all-cause mortality, myocardial infarction and stroke [6]. More than half the women who are diagnosed with MetS in early pregnancy develop major pregnancy complications including preeclampsia and gestational diabetes [7]. Maternal cardiovascular risk factors including hypertension, hyperlipidaemia and obesity have been shown to be associated with increased prevalence of CVD risk factors in their children [8, 9]. Therefore, constituents of MetS have adverse outcomes for both women and children. Although the association between major pregnancy complications and later life CVD in women is well known, at present there is paucity of literature on the progression of early pregnancy CVD risk factors postpartum. Hence, we aimed to study the association between early pregnancy cardio-metabolic risk factors and the prevalence of metabolic syndrome (MetS), a surrogate marker of risk for CVD at 10 years postpartum. Our primary aim was to compare the prevalence of MetS 10 years postpartum among women who had one or more pre-defined conventional cardio-metabolic risk factor and those who had no risk factor at 15 ± 1 weeks’ gestation during the first pregnancy. The secondary aim was to compare individual cardio metabolic risk factors including, obesity, raised blood pressure, increased fasting blood glucose, adverse lipid profile, and the homeostatic model of insulin resistance (HOMA-IR) 10 years postpartum, among women who had one or more pre-defined cardio metabolic risk factor and those who had no risk factor at 15 ± 1 weeks’ gestation during the first pregnancy. As an exploratory aim, we assessed the association between major pregnancy complications in first pregnancy and the prevalence of MetS 10 years postpartum among women.

## Materials and methods

The participants of this study were women who were recruited to the SCreening fOr Pregnancy Endpoints (SCOPE) and attended a postpartum CVD risk screening assessment 10 years later. The SCOPE study (www.scopestudy.net) is an international, multicentre prospective cohort study that was conducted with the aim of developing screening tests to predict preeclampsia, small for gestational age (SGA) infants and spontaneous preterm birth (sPTB) across different populations. Details of the SCOPE study are available in previous publications [10, 11]. In brief, nulliparous women with singleton pregnancies were recruited to the SCOPE study at 15 ± 1 weeks’ gestation between November 2004 and February 2011 in Adelaide, Australia; Auckland, New Zealand; Manchester, Leeds, and London, United Kingdom; and Cork, Ireland (n = 5628). Those considered at high risk of preeclampsia, SGA infants and sPTB because of underlying medical conditions (including known preexisting chronic hypertension on hypertensive medication or having blood pressure > 160/100 mmHg at 15 ± 1 weeks’ gestation), gynecological history, or three or more miscarriages or terminations of pregnancy or couples who received medical or surgical interventions that could modify pregnancy outcome were not eligible. At the 15 ± 1 weeks’ visit, detailed information was collected including demography; medical and family history; smoking, alcohol and recreational drug use and maternal birthweight by research midwives who interviewed the women. The reported birthweights of women were verified by checking medical records when possible. At the 15 ± 1 weeks’ visit, physical measurements including height, weight, and blood pressure were obtained. Height was measured (without shoes) using a stadiometer (SECA, Germany) and weight was measured without shoes or wearing a jacket using a digital scale (SECA, Germany). Blood pressure was measured using a mercury sphygmomanometer (Reister, Germany). Non fasting blood glucose and lipids were also measured at 15 ± 1 weeks’ gestation. A research midwife obtained data on pregnancy outcome and infant measurements within 72 hours of birth. A total of 1164 women were recruited to the Adelaide SCOPE cohort between 2005–2008.

### Definitions of pregnancy outcomes

Gestational hypertension was defined as systolic blood pressure ≥ 140 mm Hg and/or diastolic blood pressure ≥ 90 mm Hg on two or more measurements 6 hours apart after 20 weeks’ gestation [12]. Preeclampsia was defined using the revised International Society for the Study of Hypertension in Pregnancy definition of gestational hypertension or postpartum hypertension with proteinuria (24-hour urinary protein of 300 mg or spot urine protein/creatinine ratio of ≥ 30 mg/mmol creatinine or urine dipstick protein ≥ ++) or any multisystem complication of preeclampsia or utero placental dysfunction as evidenced by intrauterine growth restriction [12]. An infant with a birthweight below the 10^th^ customized centile adjusted for maternal height, weight, parity and ethnicity, gestational age at delivery, and infant sex was classified as small for gestational age [13]. Birth of an infant prior to completion of 37 weeks’ gestation due to spontaneous preterm labour or preterm premature rupture of membranes was classified as sPTB. Gestational Diabetes Mellitus (GDM) was defined using the World Health Organization classification as fasting glucose ≥ 5.1 mmol/L or a 2-hour level of ≥ 8.5 mmol/L following an oral glucose tolerance test [14]. Uncomplicated pregnancy was defined as a pregnancy with no antenatal medical or obstetric complications and resulting in the delivery of an appropriately grown, healthy baby at ≥ 37 weeks’ gestation. Since all major pregnancy complications including preeclampsia, SGA pregnancy, sPTB and GDM have been shown to confer similar risks for later life CVD [3], we have included all these complications in one group as “complicated pregnancies”

### SCOPE follow up study

The Human Research Ethics Committees of the University and hospitals [HREC/15/WCHN/192 and HREC/14/TQEH/277] granted ethics approval of the SCOPE follow-up study. All women who participated in the SCOPE study consented to be contacted for future follow-up studies. Between 2015–2018, SCOPE women were invited to take part in cardiovascular risk screening 10 years after the first pregnancy. The women were contacted using the phone numbers provided during the initial SCOPE study and by using new contact information provided to the hospital after the SCOPE study. All women provided written informed consent. A total of 273 women attended the follow up visit. This study includes data from 141 women who underwent the additional blood investigations. At the 10 year follow up visit, data were collected on demography, parity, medical history and smoking at an interview with a member of the research team. The Index of Relative Socioeconomic Disadvantage (IRDS) was used as a measurement of a woman’s socioeconomic status [15]. The IRDS is derived from the postcode and takes into account data on occupation, income and educational level from a specific area and provides a score between 1 and 8 with a lower score reflecting greater disadvantage. Physical activity was measured by the International Physical Activity Questionnaire Long Form for English (IPAQ). Quality of life was assessed using the Short Form 12 (SF-12) questionnaire. Psychosocial measures were assessed by the General Anxiety Disorder (GAD-7) and Patient Health Questionnaire (PHQ-9). Height was measured and weight was measured to the nearest 0.1 kg using the TANITA SC-330 (Tokyo, Japan) bio-impedance scale, which also calculates BMI. Haemodynamic profile was assessed using the USCOM BP+ and USCOM 1A ultrasound machines (USCOM, Brisbane, Australia). A fasting blood sample was taken to measure glucose, insulin and lipids. All blood tests were done at a SA Pathology laboratory (sapathology.sa.gov.au).

### Stratification of cardio-metabolic risk factors at 15 ±1 weeks’ gestation during the first pregnancy

We classified women who attended the 10 year follow up according to the presence or absence of pre-defined cardio metabolic risk factors at 15 ±1 weeks’ gestation during the first pregnancy (BMI ≥ 30kg/m^2^, systolic blood pressure ≥ 130mmHg, diastolic blood pressure ≥ 85mmHg, total cholesterol ≥5.5mmol/L, triglycerides ≥1.7mmol/l, HDL cholesterol < 1.2mmol/l, random plasma glucose ≥5.6 mmol/L and smoking) [16].

### Primary outcome

metabolic syndrome (diagnosed using the International Diabetes Federation guidelines as [16]): the presence of central obesity, defined as waist circumference with ethnicity specific values and/or a body mass index (BMI) >30kg/m^2^, plus any two of the following four factors: raised triglycerides: ≥1.7 mmol/L or specific treatment for this lipid abnormality; reduced HDL cholesterol: <1.2 mmol/L or treatment for this lipid abnormality; raised blood pressure: systolic BP ≥130, or diastolic BP ≥85 mm Hg, or treatment of previously diagnosed hypertension; raised random plasma glucose: ≥5.6 mmol/L, or previously diagnosed type 2 diabetes.

### Secondary outcomes

previously diagnosed hypertension or a blood pressure of ≥130/85 mmHg (detected after a 20 minute rest and a second confirmed reading 1 hour later); body mass index ≥ 30 kg/m^2^; fasting blood glucose ≥5.6 mmol/l, HbA1c > 6.5% or previously diagnosed type 2 diabetes; total cholesterol >5.5 mmol/L; triglycerides ≥1.7 mmol/L; HDL <1.2mmol/L, HOMA-IR was calculated as fasting plasma glucose (mmol/l) X fasting plasma insulin (IU/l)/22.5. HOMA-IR score above 3.0 was classified as high [17].

### Statistics

Statistical analyses were performed using SPSS version 28 (IBM, Armonk, New York, USA). Data from women who had one or more cardio metabolic risk factor at 15 ± 1 weeks’ gestation during the first pregnancy were compared with data from women who had no cardio metabolic risk factor at 15 ± 1 weeks’ gestation during the first pregnancy. For categorical variables, Chi-square test was used to compare the groups and the Fisher’s exact test was used when the expected frequencies were below five. For continuous variables, student’s *t*-test was used. Binary logistic regression was used to calculate risk for MetS among women who had ≥1 cardio metabolic risk factor at 15 ± 1 weeks’ gestation during the first pregnancy compared to those who had no risk factor adjusting for age and smoking status at the 10 year follow up visit. These two confounders were selected *apriori* based on previous literature. The number of covariates that were included in the model were restricted by the sample size.

## Results

The socio-demographic characteristics of the participants according to the presence or absence of cardio metabolic risk factors at 15 ± 1weeks’ gestation during the first pregnancy are shown in Table 1. Of the 141 women who completed all assessments and investigations, 41 (29.1%) did not have any cardio-metabolic risk factors and 100 (70.9%) had ≥ 1 risk factor at 15 ±1 weeks’ gestation during the first pregnancy. Of the 100 women who had ≥ 1 risk factor at 15 ±1 weeks’ gestation, 26 (26%) had MetS during pregnancy. Women who had ≥1 cardio metabolic risk factor during first pregnancy were ~2 years older than women who did not have any risk factor (37.7 years vs 35.1 years, Table 1). No significant differences were detected between the two groups in other socio-demographic characteristics (Table 1).

**Table 1:** Characteristics of the study population at 10 years postpartum.

Characteristic	No risk factor in first pregnancy (n = 41)	≥ 1 risk factor in first pregnancy (n = 100)	P value
Age	35.1 ± 4.1	37.7 ± 5.3	0.004
Gravidity	2 (2, 3)	2 (2, 4)	0.5
SEI	27 (25)	30 (12)	0.9
GAD-7 score	2 (0, 5)	2 (1, 5)	0.4
PHQ-9 score	3 (1, 6)	3 (2, 6)	0.7
Hours of exercise per week	3.3 ± 2.4	2.6 ± 1.7	0.15
Family history of hypertension	27 (65.9%)	51 (51%)	0.11
Family history of type 2 diabetes mellitus	15 (36.6%)	45 (45.5%)	0.33
Family history of ischaemic heart disease	16 (39%)	39 (39%)	0.99

GAD-7, General Anxiety Disorder; PHQ-9, Patient Health Questionnaire; SES, Socioeconomic index; results are number (%), mean ± SD or median and inter quartile range. Categorical variables were compared using Chi-square test and the Fisher’s exact test. Continuous variables were compared using student’s *t*-test.

### Risk for metabolic syndrome 10 years postpartum based on presence or absence of cardiometabolic risk factors during early first pregnancy

Compared to women who did not have any cardio metabolic risk factor at 15 ± 1 weeks’ gestation during the first pregnancy, those who had ≥ 1 risk factor were 4.8 times more likely to have MetS 10 years postpartum (OR = 4.8 (95% CI 1.6–14.5, p = 0.005)). This result remained significant after adjusting for age and smoking status (aOR = 5.5, 95% CI 1.8–17.3, p = 0.004, Table 2).

**Table 2:** Risk for metabolic syndrome 10 years postpartum based on presence or absence of cardio metabolic risk factors during early first pregnancy.

MetS 10 years postpartum	No risk factor in early pregnancy (n = 41)	≥ 1 risk factor in early pregnancy (n = 100)	OR (95% CI)	aOR (95% CI)
No MetS n (%)	37 (90.2)	66 (66.0)	1	1
MetS n (%)	4 (9.8)	34 (34.0)	4.8 (1.6–14.5)	5.5 (1.8–17.3)

aOR, adjusted for age and smoking status at 15 ± 1 weeks’ gestation during first pregnancy.

### Individual risk factors for CVD 10 years postpartum based on presence or absence of cardio metabolic risk factors during early first pregnancy

Prevalence of individual CVD risk factors 10 years postpartum were higher among women who had ≥ 1 cardio-metabolic risk factor during the first pregnancy (Table 3). Women who had ≥ 1 risk factor at 15 ± 1 weeks’ gestation during the first pregnancy were significantly more likely to be obese (p = 0.001), have high total cholesterol levels (p <0.001) or have increased insulin resistance (p <0.001) 10 years later compared to women who had no risk factor at 15 ± 1 weeks’ gestation during the first pregnancy (Table 3).

**Table 3:** Individual risk factors for CVD at 10 years postpartum among women who had one or more CVD risk factor and those who did not have any risk factor in early first pregnancy.

CVD Risk factor 10 years postpartum	No risk factor in first pregnancy (n = 41)	≥ 1 risk factor in first pregnancy (n = 100)	P value
Elevated SBP	4 (9.8%)	14 (14%)	0.35
Elevated DBP	4 (9.8%)	14 (14%)	0.35
Obesity	10 (24.4%)	56 (56%)	0.001
Increased total cholesterol	1 (2.4%)	42 (42%)	<0.001
Increased triglycerides	2 (4.9%)	16 (16%)	0.058
Low HDL cholesterol	8 (19.5%)	32 (32%)	0.13
Increased fasting plasma glucose	1 (2.4%)	12 (12%)	0.07
Smoking	3 (7.3%)	10 (10%)	0.44
High HOMA-IR*	3 (7.5)	33 (34.7)	<0.001

Results are number (%), *Insulin levels were available from 135 women. SBP, systolic blood 533 pressure; DBP, diastolic blood pressure; HDL, high density lipoprotein cholesterol, HOMA-IR, homeostatic model of insulin resistance. Categorical variables were compared using Chi-square test and the Fisher’s exact test. Continuous variables were compared using student’s *t*-test.

### Risk for metabolic syndrome 10 years postpartum based on presence or absence of cardio metabolic risk factors during early first pregnancy and presence or absence of pregnancy complications

Of the women who had no cardio metabolic factor 237 at 15 ± 1 weeks’ gestation during the first pregnancy, 63.5% did not develop any pregnancy complication and were not diagnosed with MetS 10 years postpartum (Table 4). Of the women who had ≥ 1 cardio metabolic risk factor at 15 ± 1 weeks’ gestation during the first pregnancy, 39% did not develop any pregnancy complication and were not diagnosed with MetS 10 years postpartum (Table 4). Approximately 27% of women in both groups developed at least one pregnancy complication but were not diagnosed with MetS 10 years later (Table 4). 13% of women who had ≥ 1 risk factors at 15 ± 1 weeks’ gestation during the first pregnancy compared to 2.4% who had no risk factor did not develop any pregnancy complication but were diagnosed with MetS 10 years postpartum (Table 4). 21% of women who had ≥ 1 risk factor at 15 ± 1 weeks’ gestation during the first pregnancy compared to 7.3% who had no risk factor developed one or more pregnancy complications and were also diagnosed with MetS 10 years postpartum (Table 4).

**Table 4:** Prevalence of pregnancy complications and or metabolic syndrome stratified by cardio metabolic risk factors during early first pregnancy.

Risk factor in first pregnancy	No PC + No MetS	PC + No MetS	No PC + MetS	PC + MetS
No risk factor (n = 41)	26 (63.5%)	11 (26.8%)	1 (2.4%)	3 (7.3%)
≥ 1 risk factor (n = 100)	39 (39.0%)	27 (27.0%)	13 (13.0%)	21 (21.0%)

Results are number (%); PC, pregnancy complication; MetS, metabolic syndrome.

## Discussion

This study demonstrates that cardio metabolic risk factors during early first pregnancy associate with the risk for MetS 10 years postpartum. Our results show that women who have one or more cardio metabolic risk factor at 15 ± 1 weeks gestation during the first pregnancy have 5.5 times higher odds of being diagnosed with MetS 10 years later compared to women who have no cardio metabolic risk factor at 15 ± 1 weeks gestation during the first pregnancy.

The association between MetS and risk for cardiovascular disease has been examined in numerous epidemiologic studies. Individuals with MetS, compared to those without are known to have increased mortality from all causes and CVD and have increased incidence of CVD [18]. The relative risk of cardiovascular disease associated with MetS is also known to be higher in women compared with men [18]. In our cohort of young women, 34% of women who had ≥1 cardio metabolic risk factor during the first pregnancy had MetS by the age of 37.7 ± 5.3 years, demonstrating an increased risk for CVD at a relatively young age.

Cardiovascular diseases including coronary heart disease and stroke are the leading non-communicable diseases globally and accounted for an estimated 17.8 million deaths in 2017 [19]. Traditionally considered a disease that predominantly affects men, CVD remains the number one mortality among women around the world [2]. Regardless of the age at diagnosis, within one year of a first acute myocardial infarction, more women compared to men die (26% of women vs 19% of men) and within 5 years of a first acute myocardial infarction more women than men die (47% of women vs 36% of men), have heart failure or experience a stroke [20].

In Australia, cardiovascular diseases (predominantly ischaemic heart disease and stroke) resulted in 43,477 deaths (27%) and 42.6 deaths per 100,000 among females in 2017 [21]. Based on the 2011–12 Australian National Health Measures Survey, ~14% of Australian women aged 45–74 (522,000 women) were at high absolute risk of a future CVD event such as myocardial infarction, stroke or death from CVD over the following 5 years. A further 2.9% (108,000 women) were estimated to be at moderate risk of CVD [22]. Of the women at high absolute risk who already had CVD, only 48% were receiving lipid-lowering medication, 63% were receiving blood pressure-lowering medication, and only 34% were receiving both blood pressure and lipid-lowering medication [22] demonstrating the importance of early identification and targeted interventions in those at high risk.

With the goal of reducing CVD, in 2010 the American heart Association established a simplified 7-item tool including smoking status, physical activity, healthy dietary intake, body mass index, blood pressure, total cholesterol and fasting plasma glucose to promote ideal cardiovascular health [23]. Since then, many studies have assessed the association between ideal cardiovascular health metrics and risk for CVD. A recent meta-analysis of nine prospective cohort studies involving 12,878 participants showed that achieving the greatest ideal cardiovascular health metrics was associated with lower risk of all-cause mortality (RR 0.55; 95% CI 0.37–0.80), cardiovascular mortality (RR 0.25; 95% CI 0.10–0.63), cardiovascular disease (RR 0.20;95% CI 0.11–0.37), and stroke (RR 0.31; 95% CI 0.25–0.38) [24]. The above results are indicative of the importance of management of modifiable CVD risk factors.

In our study obesity, total cholesterol, and high HOMA-IR index were significantly more prevalent among women who had ≥1 cardio metabolic risk factor during first pregnancy compared to women who had no risk factor (Table 3). The high prevalence of obesity (56%), elevated total cholesterol (42%) and insulin resistance (34.7%) among these young women in their late 30’s is of significant concern.

According to 2019 data from the Australian Institute of Health and Welfare, in Australia, among the major CVD risk factors, only smoking levels have declined substantially in recent decades [25]. The prevalence of smoking among Australian women aged 18 years and over decreased from 24% in 1989–90, to 11% in 2017–18. However, other risk factors including overweight and obesity among women have increased from 49% in 1995 to 60% in 2017–2018 [25]. In our study, of women who had ≥1 cardio metabolic risk factor during first pregnancy, obesity was detected at 15 ± 1 weeks’ gestation during the first pregnancy among 40% of women and this percentage had increased to 56% at 10 years postpartum demonstrating that pregnancy provides a good opportunity to identify women at risk of CVD.

The consistent evidence that links major pregnancy complications with later life CVD has resulted in some countries recommending postpartum follow up of women who experience gestational hypertensive diseases and gestational diabetes mellitus. However, no universal guidelines exist on the ideal time to commence follow up and no follow up is available for women who experience other major pregnancy complications. There is also debate as to whether pregnancy complications result in an adverse cardio metabolic profile or whether women who embark on a pregnancy with pre-existing CVD risk factors experience pregnancy complications and subsequent CVD.

Our findings show that 64% of women who have no cardio metabolic risk factor in early first pregnancy have uncomplicated first pregnancies and maintain healthy cardio metabolic metrics 10 years postpartum. Of women who have ≥ 1 cardio-metabolic risk factor during first pregnancy, 13% do not experience major pregnancy complications during the first pregnancy but have MetS 10 years later and 21% experience a major pregnancy complication during the first pregnancy and also have MetS 10 years later. These findings suggest that basic screening for CVD at the booking visit in early pregnancy may identify women at risk of later life CVD. Based on the eligibility criteria, women with BMI ≥ 35kg/m2, those with pre-existing hypertension, type 2 diabetes mellitus and high blood pressure at the booking visit were not recruited to the SCOPE study. Therefore, the actual number of women with risk factors for CVD in early pregnancy in the study population would be higher than reported in this study.

Consistent with our findings, data from 3510 women from the Generation R study demonstrated that early pregnancy lipids associated with lipid levels six years postpartum, independent of pregnancy complications. Gestational triglycerides and remnant cholesterol in the highest quartile and HDL cholesterol in the lowest quartile were associated with the highest risk for metabolic syndrome, independent of smoking and BMI [26].

We do acknowledge the limitations in our study. From a cohort of 1161 women who took part in the SCOPE study in Adelaide, we were only able to recruit 273 women for the 10 year follow up study and only 141 completed the blood tests, therefore, some degree of selection bias would exist. The main reason for the large number of women being lost to follow up was due to women being not contactable using the phone numbers provided during the initial SCOPE study, changing place of residence and not being in close proximity to the recruitment hospital and moving interstate. However, as reported in a previous publication, women who participated in the follow up were of a higher socio-economic background than the ones that did not participate [27]. Therefore, considering that adverse cardio metabolic parameters are more prevalent among the lower socio-economic strata, we would expect even a higher prevalence of CVD risk factors including MetS among the non-respondents. Many genetic, environmental and lifestyle factors affect both pregnancy complications and metabolic syndrome. We could not include many potential confounders in our models due to non-availability of some data and due to the sample size. However, the main strength of this study is that the SCOPE pregnancy cohort was a prospective, well characterised cohort. A post-hoc power calculation was performed using G*Power version 3.1 (Post-hoc Z-tests for Logistic regression, S1 Fig) [28]. Using an odds ratio of 5.5, a total of 141 participants and prevalence of metabolic syndrome in 27% of the study population, this study has 99% power to detect the differences between the groups. A two-tailed alpha level of 0.05 was used, and a Binomial distribution for a predictor variable with 70% of women having ≥1 risk factor in early pregnancy.

## Conclusion

Overall, this study shows that cardio metabolic risk factors that can easily be detected at the booking visit in the first pregnancy may be useful in identifying young women at risk of future cardiovascular disease.

## Supporting information

S1 Fig Power calculation. (PNG)
