# Supplementary figures and images for: Early pregnancy cardio metabolic risk factors and the prevalence of metabolic syndrome 10 years after the first pregnancy
Source: PLoS One. 2023 Jan 20;18(1):e0280451. doi: 10.1371/journal.pone.0280451 (PMC9858479; doi:10.1371/journal.pone.0280451)

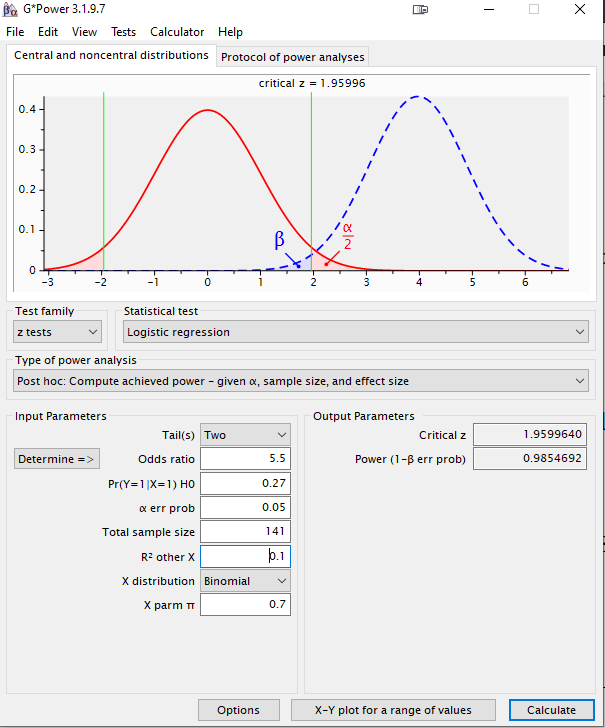

Supplement: S1 Fig — (PNG) [file pone.0280451.s001.png]
